# The Association between *Helicobacter pylori* Seropositivity and Bone Mineral Density in Adults

**DOI:** 10.1155/2022/2364666

**Published:** 2022-04-04

**Authors:** Jinke Huang, Zhihong Liu, Jinxin Ma, Jiali Liu, Mi Lv, Fengyun Wang, Xudong Tang

**Affiliations:** ^1^Department of Gastroenterology, Xiyuan Hospital, China Academy of Chinese Medical Sciences, Beijing, China; ^2^Department of Gastroenterology, Peking University Traditional Chinese Medicine Clinical Medical School (Xiyuan), Beijing, China; ^3^China Academy of Chinese Medical Sciences, Beijing, China

## Abstract

**Objectives:**

Current evidence on the associations between *Helicobacter pylori* (*H. pylori*) infection and bone mineral density (BMD) is conflicting. Therefore, a nationally representative sample of adults was analyzed to investigate the associations of *H. pylori* seropositivity and BMD in this study.

**Methods:**

A retrospective cross-sectional study was conducted with 2555 subjects aged 40-85 years in the US National Health and Nutrition Examination Survey (NHANES) 1999–2001. Multivariable logistic regression models were performed to evaluate the associations between *H. pylori* seropositivity and BMD. Subgroup analyses stratified by sex, age, race, and body mass index (BMI) were performed.

**Results:**

No association was found between *H. pylori* seropositivity and BMD (*β* = 0.006, 95% CI: -0.003 to 0.015, *P* = 0.177). In the subgroup analyses stratified by age, a positive association was observed between the *H. pylori* seropositivity and total BMD among subjects aged 40-55 years (*β* = 0.018, 95% CI: 0.004 to 0.033, *P* = 0.012); in the subgroup analyses stratified by sex, a positive association was observed between the *H. pylori* seropositive and total BMD in male (*β* = 0.019, 95% CI: 0.007 to 0.032, *P* = 0.003); in the subgroup analyses stratified by age and sex, the total BMD was higher in men aged 40-55 years with *H. pylori* seropositive than those with *H. pylori* seronegative (*β* = 0.034, 95% CI: 0.013 to 0.056, *P* = 0.002).

**Conclusions:**

In conclusion, no association between *H. pylori* seropositive and total BMD was demonstrated among most middle-aged and elderly adults. *H. pylori* infection may not be one key factor in the loss of BMD.

## 1. Introduction

*Helicobacter pylori* (*H. pylori*) is the most common chronic bacterial colonizing the human stomach. *H. pylori* infection is prevalent [1], with a prevalence of approximately 35.6% in the United States [2]. *H. pylori* infection has been well known to be associated with a variety of gastric diseases, including chronic gastritis, peptic ulcers, gastric cancer, and mucosa-associated lymphoid tissue lymphoma [3]. Furthermore, several extra gastric disorders have also been proven to be associated with *H. pylori* infection, such as metabolic, neurological, and cardiovascular diseases [4].

Osteoporosis is a silent health problem characterized by deterioration of bone structure due to low bone mineral density (BMD) and disruption of bone homeostasis [5]. As one of the most common metabolic bone diseases worldwide, osteoporosis mostly affects middle-aged and elderly populations [6]. Patients with osteoporosis are susceptible to bone fragility and osteoporotic fractures, and the occurrence of these fractures affects morbidity, mortality, and quality of life, making osteoporosis a growing health and health-economic problem worldwide [7, 8]. Therefore, understanding the risk factors is essential for the prevention, early diagnosis, and management of osteoporosis.

It is reported that *H. pylori* infection can induce inflammatory and immune reactions in individuals, which may modulate bone turnover [9]. However, evidence for the association between *H. pylori* infection and BMD is limited and controversial [10, 11]. Therefore, to investigate the association between *H. pylori* seropositivity and BMD, a population-based sample from the National Health and Nutrition Examination Survey (NHANES) was analyzed in this study.

## 2. Materials and Methods

### 2.1. Study Population

**Figure 1:**
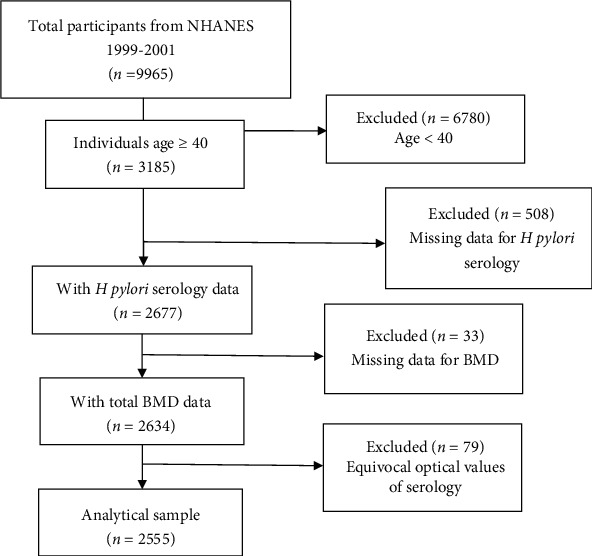
The sample selection flow chart.

The NHANES is a representative survey of the national population of US, providing multitudinous information about the nutrition and health of the general US population using a complex, multistage, and probability sampling design [12]. Data for this study was obtained from the 1999–2001 continuous cycle of the US NHANES dataset. The number of subjects in this cycle was 9965. After excluding subjects without information on laboratory and demographic variables, 2555 subjects were finally included for analyses. The sample selection flow chart is presented in Figure 1.

### 2.2. Variables

In this study, the dependent variable was *H. pylori* seropositivity, and the targeted independent variable was total BMD. *H. pylori* seropositivity was measured by the Wampole Laboratories *H. pylori* IgG Enzyme-Linked Immunosorbent Assays (ELISA). For each specimen, immune status ratio values of >1.1 and <0.9 were considered as seropositive and seronegative, respectively, whereas 0.9–1.1 were an equivocal value [13]. Subjects with equivocal values were excluded to prevent misleading statistical outcomes in this study. The measurements of total BMD were determined by DEXA scans. For covariates, sex, race, educational level, physical activity, body mass index (BMI), smoking behavior, and other disease status were used as categorical variables; age, poverty to income ratio, days drink in year, serum uric acid, total calcium, blood urea nitrogen, serum creatinine, total cholesterol, bone alkaline phosphatase, and dietary calcium intake were used as continuous variables. More detailed information on *H. pylori* seropositivity, total BMD, and the covariates is publicly available at http://www.cdc.gov/nchs/nhanes/.

### 2.3. Statistical Analysis

The design of complex sampling strategies and appropriate weight were incorporated in all analyses. Weighted multivariate linear regression models were performed to evaluate the associations between *H. pylori* seropositivity and total BMD. The other variables were considered potential effect modifiers. For continuous variables, the weighted linear regression model was used to calculate the differences among different groups. For categorical variables, the weighted chi-square test was used. All analyses were conducted in R (http://www.R-project.org, The R Foundation) and EmpowerStats software (http://www. http://empowwerstats.com/, X&Y Solutions, Inc., Boston, MA).

## 3. Results

### 3.1. Characteristics of Included Subjects

A total of 2555 subjects were included in final analyses, of which 1263 (49.43%) subjects were *H. pylori* seronegative and 1292 (50.57%) subjects were *H. pylori* seropositive. In these two groups, race, educational level, income poverty ratio, physical activity, days drink in year, smoking behavior, diabetes status, serum creatinine, bone alkaline phosphatase, dietary calcium intake, and total BMD were significantly different (*P* < 0.05). More details are presented in Table 1.

### 3.2. Association between H. pylori Seropositivity and Total BMD

#### 3.2.1. Multiple Regression Model

Three weighted univariate and multivariate linear regression models were constructed: model I, unadjusted; model II, age, sex, and race that were adjusted; and model III, covariates presented in Table 1 that were adjusted. In the unadjusted model, a negative association was found between *H. pylori* seropositivity and total BMD (*β* = −0.015, 95% CI: -0.025 to -0.006, *P* = 0.001). However, after variable adjustments, the association between *H. pylori* seropositivity and total BMD was not significant in model II and model III. Details are presented in Table 2.

#### 3.2.2. Subgroup Analyses

In the subgroup analyses stratified by age, a positive association was observed between the *H. pylori* seropositivity and total BMD among subjects aged 40-55 years (*β* = 0.018, 95% CI: 0.004 to 0.033, *P* = 0.012); however, the total BMD was not related to *H. pylori* seropositivity in other groups. In the subgroup analyses stratified by sex, a positive association was observed between the *H. pylori* seropositive and total BMD in male (*β* = 0.019, 95% CI: 0.007 to 0.032, *P* = 0.003); however, the total BMD was not related to *H. pylori* seropositivity in female. In the subgroup analyses stratified by race and BMI categories, no association was found between *H. pylori* seropositivity and total BMD. Details are presented in Table 2.

In the subgroup analysis by age and sex, a positive association was observed between the *H. pylori* seropositivity and total BMD in male aged 40–55 years (*β* = 0.034, 95% CI: 0.013 to 0.056, *P* = 0.002); however, no association was found between *H. pylori* seropositivity and total BMD in female aged 40–55 years. Moreover, in the groups of age over 55 years, no association was found between *H. pylori* seropositivity and total BMD neither male nor female. Details are presented in Table 3.

## 4. Discussion

The purpose of this study was to explore the associations between *H. pylori* seropositivity and total BMD using the data from NHANES. In summary, no association was found between *H. pylori* seropositivity and total BMD among most middle-aged and elderly adults. However, in the subgroup analyses stratified by age, a positive association was observed between the *H. pylori* seropositivity and total BMD among subjects aged 40-55 years; in the subgroup analyses stratified by sex, a positive association was observed between the *H. pylori* seropositive and total BMD in male; in the subgroup analyses stratified by age and sex, the total BMD was higher in men aged 40-55 years with *H. pylori* seropositive than those with *H. pylori* seronegative.

Osteoporosis, as one of the metabolic bone diseases, is characterized by constant loss of BMD. It is important to understand the risk factors for BMD loss, which can help in the prevention, early diagnosis, and management of osteoporosis. *H. pylori* has been coevolved with humans over 50,000 years. Infection with *H. pylori* is a common risk factor for susceptibility to metabolic diseases; however, the association between *H. pylori* infection and BMD is limited and controversial. In most studies [14–21], no association between *H. pylori* infection and BMD or osteoporosis was observed, which is consistent with our observation. Recently, a meta-analysis including 1321 adults without other causes of osteoporosis or pathological bone disease at baseline showed that *H. pylori* infection was not associated with osteoporosis (OR = 1.49, 95% CI: 0.88 to 2.55) [11]. However, another pooled study [10] including 9655 subjects came to the opposite conclusion that *H. pylori* infection was associated with increased odds of osteoporosis (OR = 1.39, 95% CI: 1.13 to 1.71). Notably, as acknowledged by the authors, the reference value of the results needs to be further validated due to the heterogeneity of the included studies. Thus, heterogeneity between these studies, including differences in the study design, study simple, and the controlled confounding variables, may explain the controversial findings. In this study, a nationally representative sample of US was used, so the findings are highly relevant to the whole population. Additionally, we further performed subgroup analyses for more appropriate representation of the dataset as recommended by the STROBE statement [22], and a special group was found; that is, a positive association was found between *H. pylori* seropositive and total BMD in male aged 40–55 years. As recently reported, sex and age are also predictors of *H. pylori* infection and BMD [9]. Furthermore, an interesting finding was reported; that is, 63.6% studies conducted in Eastern countries have observed an association between *H. pylori* infection and BMD status, whereas only 22.2% studies conducted in Western countries have observed such association [9]. The difference in the prevalence of *H. pylori* infection (about 30% in developed countries and up to 80% in developing countries) seems to contribute to the understanding of these findings [20].

The mechanism by which *H. pylori* infection increases the risk of osteoporosis and fracture remains to be elucidated in detail. Based on the evidence available to date, several potential mechanisms may underlie the association between *H. pylori* infection and osteoporosis. First, proinflammatory cytokines can act on mesenchymal stem cells and osteoclast precursors to enhance osteoclast-mediated bone resorption [23]. It was reported that high levels of circulating inflammatory markers were associated with increased bone loss or increased fracture risk [24, 25]. Therefore, it is likely that *H. pylori* increases the risk of osteoporosis by promoting an inflammatory response to produce proosteoclastogenic cytokines such as TNF*α*, IL-1, IL-6, and IL-8 [23]. Second, *H. pylori* infection was associated with reduced levels of estrogen, total estradiol, free estradiol, and bioavailable estradiol in both genders [26]. It was found that decreased estrogen production was associated with a sustained increase in the spontaneous secretion of osteoclastogenic cytokines by T cells, mononuclear cells, and bone marrow stromal cells, leading to net bone loss with increased bone resorption and decreased bone formation [27]. Third, chronic H. pylori infection may be associated with gastric mucosal atrophy. Atrophy of the gastric mucosa can inhibit acid secretion and thus affect calcium absorption and consequently adversely affect bone mass [15]. However, a recent cross-sectional study of 268 healthy men showed that decreased bone mineral density was not associated with *H. pylori*-associated estradiol levels or gastric mucosal atrophy [21]. Similarly, the chronic use of proton pump inhibitors for treatment for gastroduodenal mucosal injury may result in low levels of gastric acid, which is believed to impair calcium solubility and lead to malabsorption, thereby exacerbating bone mineral density loss secondary to hypocalcemic hyperparathyroidism, osteoclast activation, and bone resorption [28, 29]. Overall, the current available data are equivocal, and further mechanistic studies are still necessary.

To the best of our knowledge, this is the first study to explore the association between *H. pylori* seropositive and BMD using the data from NHANES. The NHANES features a rigorous sampling design from the national population of US, high-quality research measurement, detailed quality control procedures, and a more representative population. However, limitations must be acknowledged. First, all data in NHANES are cross-sectional; this study cannot draw the causal relationship between *H. pylori* seropositive and total BMD. Second, the bias caused by other potential confounding factors that did not be adjusted in this study is not excluded. Furthermore, this study did not include the inflammation status of the subjects, which can possibly explain the relationship between *H. Pylori* seropositive and bone health.

## 5. Conclusion

In conclusion, no association between *H. pylori* seropositive and total BMD was demonstrated in our study. *H. pylori* infection may not be one key factor in the loss of BMD.

## Figures and Tables

**Table 1:** Weighted characteristics of included subjects.

	*H. pylori* seronegative (*n* = 1263)	*H. pylori* seropositive (*n* = 1292)	*P* value
Age (years)	60.493 ± 13.239	61.158 ± 12.959	0.200
Sex (%)			0.433
Male	48.219	49.768	
Female	51.781	50.232	
Race (%)			<0.001
Non-Hispanic white	67.379	27.941	
Non-Hispanic black	12.747	22.755	
Mexican American	14.727	38.854	
Other races	5.146	10.449	
Educational level (%)			<0.001
Less than high school	26.920	59.211	
High school	25.178	17.492	
College graduate or above	47.902	23.297	
Ratio of family income to poverty	6.821 ± 2.856	5.794 ± 2.568	<0.001
Body mass index (100%)			0.398
Undernutrition	2.142	1.208	
Normal	30.038	29.890	
Overweight	35.224	35.551	
Obese	32.596	33.351	
Smoking behavior (%)			0.044
None	48.614	47.988	
Past	35.154	32.198	
Current	16.231	19.814	
Days drink in year	96.090 ± 97.198	79.335 ± 78.680	<0.001
Physical activity (MET-based rank) (100%)			<0.001
0	24.149	38.313	
1	32.225	30.186	
2	16.706	11.842	
3	26.920	19.659	
Any hypertension (%)			0.197
None	57.641	55.108	
Yes	42.359	44.892	
Any diabetes (%)			<0.001
None	86.936	81.115	
Yes	13.064	18.885	
Any coronary artery disease (%)			0.774
None	94.458	94.195	
Yes	5.542	5.805	
Serum uric acid (mg/dL)	5.408 ± 1.558	5.462 ± 1.637	0.389
Total calcium (mg/dL)	9.349 ± 0.898	5.462 ± 1.637	0.255
Blood urea nitrogen (mg/dL)	15.777 ± 6.508	15.630 ± 6.294	0.563
Serum creatinine (*μ*mol/L)	1.419 ± 0.470	1.454 ± 0.515	0.070
Total cholesterol (mg/dL)	203.684 ± 42.647	204.084 ± 43.037	0.813
Bone alkaline phosphatase (mg/dL)	362.894 ± 85.213	372.056 ± 83.924	0.006
Dietary calcium intake (mg)	795.699 ± 493.174	671.269 ± 505.352	<0.001
Total bone mineral density (g/cm^2^)	1.088 ± 0.130	1.074 ± 0.129	0.007

Mean ± SD for continuous variables: *P* value was calculated by a weighted linear regression model. % for categorical variables: *P* value was calculated by a weighted chi-square test.

**Table 2:** Association of *H. pylori* seropositive and total bone mineral density.

Effect modifier	Model I (*β*, 95% CI, *P*)	Model II (*β*, 95% CI, *P*)	Model III (*β*, 95% CI, *P*)
Total	-0.015 (-0.025, -0.006) 0.001	-0.002 (-0.011, 0.007) 0.665	0.006 (-0.003, 0.015) 0.177
Age groups			
40-55 years (*n* = 977)	-0.001 (-0.016, 0.013) 0.850	0.004 (-0.011, 0.019) 0.590	0.018 (0.004, 0.033) 0.012
~70 years (*n* = 921)	-0.019 (-0.035, -0.003) 0.0188	-0.014 (-0.029, 0.001) 0.064	-0.010 (-0.025, 0.004) 0.169
~85 years (*n* = 657)	0.007 (-0.012, 0.026) 0.487	0.008 (-0.008, 0.023) 0.329	0.009 (-0.007, 0.024) 0.257
Sex			
Male (*n* = 1252)	0.002 (-0.011, 0.015) 0.746	0.013 (-0.000, 0.026) 0.056	0.019 (0.007, 0.032) 0.003
Female (*n* = 1303)	-0.031 (-0.044, -0.018) <0.001	-0.014 (-0.025, -0.002) 0.023	-0.002 (-0.014, 0.009) 0.730
Race			
Non-Hispanic White (*n* = 1212)	-0.023 (-0.039, -0.007) 0.004	-0.003 (-0.016, 0.010) 0.655	0.008 (-0.005, 0.021) 0.237
Non-Hispanic Black (*n* = 455)	-0.019 (-0.043, 0.006) 0.131	-0.018 (-0.039, 0.003) 0.100	-0.013 (-0.035, 0.008) 0.230
Mexican American (*n* = 688)	0.006 (-0.013, 0.025) 0.514	-0.004 (-0.020, 0.013) 0.672	0.003 (-0.014, 0.020) 0.703
Other races (*n* = 200)	0.018 (-0.013, 0.049) 0.256	0.024 (-0.004, 0.051) 0.089	0.015 (-0.010, 0.041) 0.244
BMI categories			
Undernutrition (*n* = 36)	-0.081 (-0.199, 0.037) 0.188	0.020 (-0.093, 0.134) 0.728	-0.000 (-0.282, 0.281) 0.997
Normal (*n* = 695)	-0.015 (-0.034, 0.004) 0.131	0.001 (-0.017, 0.018) 0.951	0.017 (-0.001, 0.035) 0.064
Overweight (*n* = 953)	-0.010 (-0.026, 0.006) 0.234	0.005 (-0.009, 0.019) 0.515	0.007 (-0.007, 0.022) 0.304
Obese (*n* = 871)	-0.023 (-0.039, -0.007) 0.0056	-0.008 (-0.022, 0.006) 0.270	-0.003 (-0.017, 0.011) 0.668

Model I: no covariates were adjusted; model II: age, sex, and race were adjusted; model III: age, sex, race, educational level, body mass index, ratio of family income to poverty, physical activity, smoking behavior, hypertension status, diabetes status, coronary artery disease status, serum uric acid, total calcium, blood urea nitrogen, serum creatinine, total cholesterol, bone alkaline phosphatase, and dietary calcium intake were adjusted.

**Table 3:** Total bone mineral density stratified by race and age.

*Helicobacter pylori* infection	Total bone mineral density (g/cm^2^) (*β*, 95% CI, *P*)		
	40-55 years	56~70 years	71~85 years
Male	0.034 (0.013, 0.056) 0.002	-0.001 (-0.022, 0.020) 0.907	0.010 (-0.014, 0.015) 0.907
Female	0.005 (-0.014, 0.023) 0.616	-0.011 (-0.031, 0.009) 0.285	0.008 (-0.014, 0.029) 0.485

Adjusted for age, sex, race, educational level, body mass index, ratio of family income to poverty, physical activity, smoking behavior, hypertension status, diabetes status, coronary artery disease status, serum uric acid, total calcium, blood urea nitrogen, serum creatinine, total cholesterol, bone alkaline phosphatase, and dietary calcium intake.

## Data Availability

The datasets analyzed during the current study are available in the NHANES repository (https://wwwn.cdc.gov/nchs/nhanes/Default.aspx).

## Abbreviations

*H. pylori*: *Helicobacter pylori*
BMD: Bone mineral density
NHANES: National Health and Nutrition Examination Survey.
